# Bis(pyridine-2-carboxyl­ato-κ^2^ *N*,*O*)copper(II)]–benzene-1,3,5-tri­carb­oxy­lic acid–water (1/2/2)

**DOI:** 10.1107/S2414314621006726

**Published:** 2021-07-09

**Authors:** Wenkai Zhang, Bingguang Zhang, Qiaozhen Sun

**Affiliations:** a Key Laboratory of Catalysis and Materials Sciences of the State Ethnic Affairs, Commission & Ministry of Education, College of Chemistry and Material Science, South-Central University for Nationalities, Wuhan 430074, People’s Republic of China; b Key Laboratory of Non-ferrous Metals of the Ministry of Education, School of Materials Science and Engineering, Central South University, Changsha, 410083, People’s Republic of China; Howard University, USA

**Keywords:** crystal structure, mixed ligands coordination polymer, copper carboxyl­ates, hydrogen bond

## Abstract

In the title complex, [Cu(C_6_H_4_O_2_N)_2_]·2C_9_H_6_O_6_·2H_2_O, the Cu^2+^ ion lies on a center of inversion and coordinates with symmetry related pyridine nitro­gen and carboxyl oxygen atoms from two pyridine-2-carb­oxy­lic acid anions, giving rise to a square-planar coordination geometry. There are weak axial bonds between Cu and an O atom of a symmetry-related trimesic acid moieties [Cu⋯O = 2.837 (2) Å] The Cu⋯O weak inter­actions and hydrogen bonds stabilize the whole structure.

## Abstract 2

The structure of a copper(II) complex is described.

## Graphical abstract

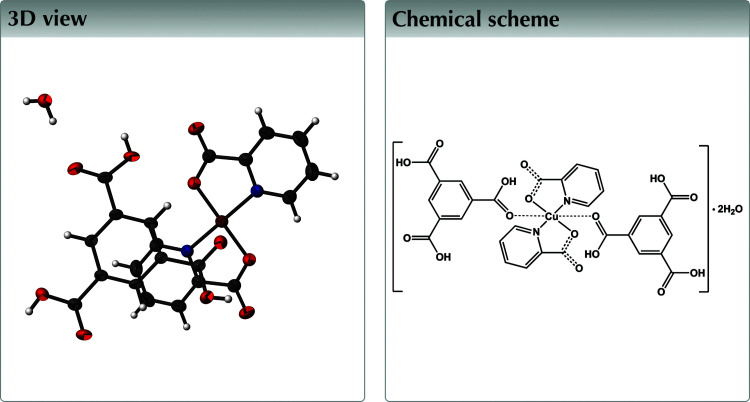

## Structure description

**Figure 1:**
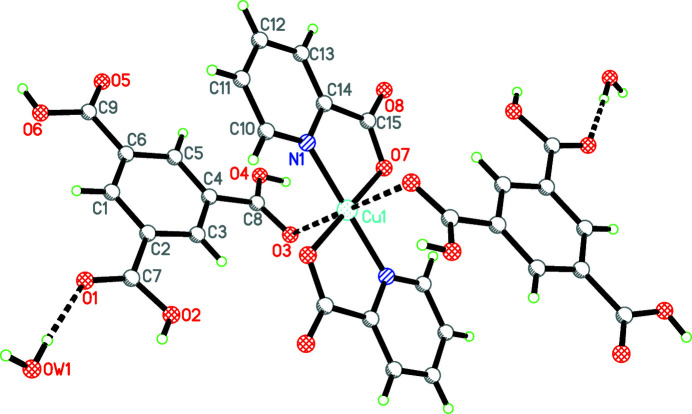
The title compound showing the atom-labelling scheme with displacement ellipsoids drawn at the 30% probability level. Unlabelled atoms are generated by the symmetry operation −*x*, −*y*, −*z*. Hydrogen bonds are shown by dashed lines

The asymmetric unit of the title compound contains half copper center, one pyridine-2-carb­oxy­lic acid anion, one BTC (benzene-1,3,5-tri­carb­oxy­lic acid) ligand and one crystal water mol­ecule (Fig. 1 ▸). The Cu^2+^ ion lies on the symmetry center and is coordinated by two symmetry-related pyridine nitro­gen atoms and two symmetry-related carboxyl oxygen atoms, giving rise to a square-planar coordination geometry. In the axial position, a very weak inter­action Cu1⋯O3 [2.837 (2) Å] is observed. Inter­estingly, the 1,4-bis­(3-pyrid­yl)-2,3-di­aza-1,3-butadiene ligand decomposed during the hydro­thermal process and is oxidized into pyridine-2-carb­oxy­lic acid. According to our earlier research, the occurrence of oxidation may be caused by excess of Cu^II^ salt, which may act as an oxidative agent to promote the formation of the carboxyl group (Sun *et al.*, 2016 ▸). Each pyridine-2-carb­oxy­lic acid anion coordinates with one Cu^2+^ ion in a bidentate *N*,*O*-chelated mode, forming a five-membered ring.

**Figure 2:**
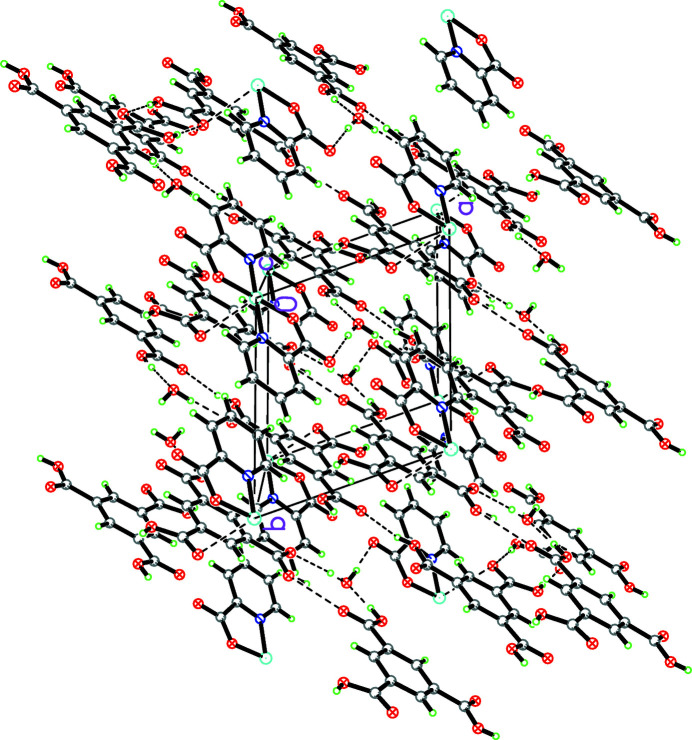
The packing of the title compound. Hydrogen bonds and Cu⋯O inter­actions are shown as dashed lines.

In the crystal, C—H⋯O and O—H⋯O hydrogen bonds (Table 1 ▸) and together with weak Cu⋯O inter­actions link the complex mol­ecules into a three-dimensional framework (Fig. 2 ▸). Although the O1⋯C9 and O6⋯C7 distances [3.002 (3)and 3.014 (3) Å, respectively] between the two symmetry-related BTC^3−^ ligands (symmetry code: −*x*, −*y*, −1 − *z*) are short, there are no π–π inter­actions because the inter-centroid distance between the two benzene rings is 5.4029 (15) Å, which is much larger than the normal π–π stacking distance of 3.3–3.8 Å. The shortest distance between the two carbon atoms (C1 and C1′) is 3.379 (4) Å. The other C⋯C distances of the two rings are longer than 3.94 Å. In addition, the distance between the centroid of one benzene ring and the C atoms of another is longer than 4.28 Å.

## Synthesis and crystallization

A mixture of trimesic acid (21 mg, 0.1 mmol), 1,4-bis­(3-pyrid­yl)-2,3-di­aza-1,3-butadiene (2-bpdb, 11 mg, 0.05 mmol) and CuCl_2_·2H_2_O (51 mg, 0.3 mmol) in 5 mL of distilled H_2_O was stirred for 10 min in air, and then the mixture was turned into a Parr Teflon-lined stainless steel vessel and heated at 160°C for 60 h. Dark-red crystals suitable for X-ray diffraction were obtained in a yield of 78% (based on CuCl_2_·2H_2_O).

## Refinement

Crystal data, data collection and structure refinement details are summarized in Table 2 ▸.

## Supplementary Material

Crystal structure: contains datablock(s) I. DOI: 10.1107/S2414314621006726/bv4039sup1.cif

Structure factors: contains datablock(s) I. DOI: 10.1107/S2414314621006726/bv4039Isup3.hkl

CCDC reference: 2092720

Additional supporting information:  crystallographic information; 3D view; checkCIF report

## Figures and Tables

**Table 1:** Hydrogen-bond geometry (Å, °)

*D*—H⋯*A*	*D*—H	H⋯*A*	*D*⋯*A*	*D*—H⋯*A*
O2—H2⋯O5^i^	0.82	1.84	2.621 (2)	158
O4—H4⋯O3^ii^	0.82	1.85	2.659 (3)	167
O6—H6⋯O*W*1^iii^	0.82	1.73	2.553 (3)	176
C10—H10⋯O7^iv^	0.93	2.66	3.121 (3)	112
C13—H13⋯O4^v^	0.93	2.61	3.454 (4)	152
O*W*1—H*W*1*A*⋯O8^vi^	0.85	1.88	2.729 (3)	174
O*W*1—H*W*1*A*⋯O7^vi^	0.85	2.66	3.259 (3)	129
O*W*1—H*W*1*B*⋯O1	0.85	2.02	2.872 (3)	175

Symmetry codes: (i) 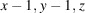 ; (ii) 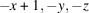 ; (iii) 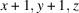 ; (iv) 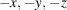 ; (v) 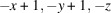 ; (vi) 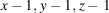 .

**Table 2:** Experimental details

Crystal data	
Chemical formula	[Cu(C_6_H_4_NO_2_)_2_]·2C_9_H_6_O_6_·2H_2_O
*M*_r_	764.05
Crystal system, space group	Triclinic, *P* 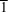
Temperature (K)	296
*a*, *b*, *c* (Å)	7.9262 (6), 8.5356 (6), 12.3629 (9)
α, β, γ (°)	107.081 (2), 90.644 (2), 108.679 (2)
*V* (Å^3^)	752.26 (10)
*Z*	1
Radiation type	Mo *K*α
μ (mm^−1^)	0.82
Crystal size (mm)	0.31 × 0.14 × 0.12

Data collection	
Diffractometer	Bruker APEXII CCD
Absorption correction	Multi-scan (*SADABS*; Bruker, 2015 ▸)
*T*_min_, *T*_max_	0.639, 0.746
No. of measured, independent and observed [*I* > 2σ(*I*)] reflections	14996, 3473, 2501
*R*_int_	0.062
(sin θ/λ)_max_ (Å^−1^)	0.651

Refinement	
*R*[*F* ^2^ > 2σ(*F* ^2^)], *wR*(*F* ^2^), *S*	0.047, 0.123, 1.06
No. of reflections	3473
No. of parameters	238
H-atom treatment	H-atom parameters constrained
Δρ_max_, Δρ_min_ (e Å^−3^)	0.41, −0.46

Computer programs: *APEX2* and *SAINT* (Bruker, 2015 ▸), *SHELXTL* (Sheldrick, 2008 ▸) and *SHELXL2014/7* (Sheldrick, 2015 ▸).

## full crystallographic data

### Crystal data

**Table:**

[Cu(C_6_H_4_NO_2_)_2_]·2C_9_H_6_O_6_·2H_2_O	*Z* = 1
*M**_r_* = 764.05	*F*(000) = 391
Triclinic, *P*1	*D*_x_ = 1.687 Mg m^−^^3^
*a* = 7.9262 (6) Å	Mo *K*α radiation, λ = 0.71073 Å
*b* = 8.5356 (6) Å	Cell parameters from 5514 reflections
*c* = 12.3629 (9) Å	θ = 3.1–26.9°
α = 107.081 (2)°	µ = 0.82 mm^−^^1^
β = 90.644 (2)°	*T* = 296 K
γ = 108.679 (2)°	Prism, red
*V* = 752.26 (10) Å^3^	0.31 × 0.14 × 0.12 mm

### Data collection

**Table:**

Bruker APEXII CCD diffractometer	2501 reflections with *I* > 2σ(*I*)
phi and ω scans	*R*_int_ = 0.062
Absorption correction: multi-scan (SADABS; Bruker, 2015)	θ_max_ = 27.6°, θ_min_ = 3.1°
*T*_min_ = 0.639, *T*_max_ = 0.746	*h* = −10→10
14996 measured reflections	*k* = −11→11
3473 independent reflections	*l* = −16→16

### Refinement

**Table:**

Refinement on *F*^2^	0 restraints
Least-squares matrix: full	Hydrogen site location: mixed
*R*[*F*^2^ > 2σ(*F*^2^)] = 0.047	H-atom parameters constrained
*wR*(*F*^2^) = 0.123	*w* = 1/[σ^2^(*F*_o_^2^) + (0.0543*P*)^2^ + 0.4521*P*] where *P* = (*F*_o_^2^ + 2*F*_c_^2^)/3
*S* = 1.06	(Δ/σ)_max_ < 0.001
3473 reflections	Δρ_max_ = 0.41 e Å^−^^3^
238 parameters	Δρ_min_ = −0.46 e Å^−^^3^

### Special details

**Table:**

Geometry. All esds (except the esd in the dihedral angle between two l.s. planes) are estimated using the full covariance matrix. The cell esds are taken into account individually in the estimation of esds in distances, angles and torsion angles; correlations between esds in cell parameters are only used when they are defined by crystal symmetry. An approximate (isotropic) treatment of cell esds is used for estimating esds involving l.s. planes.
Refinement. All of the non-hydrogen atoms were refined with anisotropic thermal displacement coefficients. Hydrogen atoms attached to the carbons were placed in their calculated position and refined with a idealized riding model. Those attached to oxygen were first located in a difference Fourier and then refined with a idealized riding model [U_iso_(H) = 1.2U_eq_(C) or 1.5 U_eq_(O)].

### Fractional atomic coordinates and isotropic or equivalent isotropic displacement parameters (Å^2^)

**Table:**

	*x*	*y*	*z*	*U*_iso_*/*U*_eq_	
Cu1	0.0000	0.0000	0.0000	0.04024 (18)	
O1	−0.1595 (2)	−0.3787 (3)	−0.56042 (16)	0.0418 (5)	
O2	−0.1580 (3)	−0.4334 (3)	−0.39491 (18)	0.0456 (5)	
H2	−0.2531	−0.5104	−0.4252	0.068*	
O3	0.2978 (3)	−0.0951 (3)	−0.08146 (16)	0.0428 (5)	
O4	0.5456 (3)	0.0969 (3)	−0.10936 (18)	0.0500 (6)	
H4	0.5797	0.0972	−0.0464	0.075*	
O5	0.5473 (3)	0.2951 (3)	−0.44481 (17)	0.0453 (5)	
O6	0.3404 (2)	0.1325 (2)	−0.59294 (15)	0.0367 (4)	
H6	0.4000	0.2025	−0.6230	0.055*	
O7	0.1869 (3)	0.1722 (2)	0.11419 (16)	0.0457 (5)	
O8	0.3797 (3)	0.4424 (3)	0.15484 (19)	0.0594 (6)	
N1	0.0395 (3)	0.1833 (3)	−0.07130 (18)	0.0344 (5)	
C1	0.1570 (3)	−0.0820 (3)	−0.4716 (2)	0.0275 (5)	
H1	0.1094	−0.1022	−0.5455	0.033*	
C2	0.0714 (3)	−0.1918 (3)	−0.4104 (2)	0.0263 (5)	
C3	0.1436 (3)	−0.1621 (3)	−0.3011 (2)	0.0294 (6)	
H3	0.0860	−0.2343	−0.2595	0.035*	
C4	0.3020 (3)	−0.0248 (3)	−0.2530 (2)	0.0281 (5)	
C5	0.3855 (3)	0.0857 (3)	−0.3140 (2)	0.0291 (6)	
H5	0.4904	0.1787	−0.2815	0.035*	
C6	0.3125 (3)	0.0574 (3)	−0.4232 (2)	0.0268 (5)	
C7	−0.0944 (3)	−0.3437 (3)	−0.4643 (2)	0.0296 (6)	
C8	0.3841 (3)	−0.0081 (3)	−0.1403 (2)	0.0318 (6)	
C9	0.4110 (3)	0.1745 (3)	−0.4883 (2)	0.0299 (6)	
C10	−0.0462 (4)	0.1813 (4)	−0.1655 (2)	0.0417 (7)	
H10	−0.1388	0.0809	−0.2067	0.050*	
C11	−0.0005 (5)	0.3247 (4)	−0.2033 (3)	0.0509 (8)	
H11	−0.0636	0.3222	−0.2680	0.061*	
C12	0.1394 (5)	0.4710 (4)	−0.1440 (3)	0.0555 (9)	
H12	0.1745	0.5675	−0.1696	0.067*	
C13	0.2268 (4)	0.4741 (4)	−0.0469 (3)	0.0479 (8)	
H13	0.3214	0.5726	−0.0056	0.058*	
C14	0.1726 (4)	0.3293 (4)	−0.0113 (2)	0.0363 (6)	
C15	0.2557 (4)	0.3172 (4)	0.0947 (2)	0.0396 (7)	
OW1	−0.4676 (3)	−0.6609 (3)	−0.69195 (17)	0.0420 (5)	
HW1A	−0.5227	−0.6321	−0.7381	0.063*	
HW1B	−0.3783	−0.5734	−0.6547	0.063*	

### Atomic displacement parameters (Å^2^)

**Table:**

	*U*^11^	*U*^22^	*U*^33^	*U*^12^	*U*^13^	*U*^23^
Cu1	0.0481 (3)	0.0303 (3)	0.0334 (3)	−0.0012 (2)	−0.0049 (2)	0.0132 (2)
O1	0.0319 (10)	0.0412 (11)	0.0375 (11)	−0.0042 (9)	−0.0068 (8)	0.0094 (9)
O2	0.0320 (11)	0.0391 (11)	0.0521 (12)	−0.0131 (9)	−0.0084 (9)	0.0227 (10)
O3	0.0393 (11)	0.0464 (12)	0.0345 (11)	−0.0023 (9)	−0.0033 (8)	0.0197 (9)
O4	0.0395 (11)	0.0524 (13)	0.0460 (12)	−0.0101 (10)	−0.0180 (9)	0.0269 (10)
O5	0.0347 (11)	0.0398 (11)	0.0472 (12)	−0.0118 (9)	−0.0043 (9)	0.0202 (9)
O6	0.0364 (11)	0.0366 (11)	0.0319 (10)	0.0004 (8)	0.0015 (8)	0.0167 (8)
O7	0.0544 (13)	0.0332 (11)	0.0388 (11)	−0.0032 (9)	−0.0106 (9)	0.0162 (9)
O8	0.0581 (14)	0.0435 (13)	0.0561 (14)	−0.0120 (11)	−0.0223 (11)	0.0188 (11)
N1	0.0386 (13)	0.0311 (12)	0.0292 (12)	0.0051 (10)	0.0015 (9)	0.0104 (9)
C1	0.0235 (12)	0.0282 (13)	0.0282 (13)	0.0060 (10)	−0.0004 (10)	0.0084 (10)
C2	0.0204 (12)	0.0231 (12)	0.0328 (13)	0.0043 (10)	0.0006 (10)	0.0085 (10)
C3	0.0253 (13)	0.0264 (13)	0.0352 (14)	0.0033 (10)	0.0021 (10)	0.0140 (11)
C4	0.0266 (13)	0.0259 (12)	0.0291 (13)	0.0048 (10)	−0.0011 (10)	0.0095 (10)
C5	0.0210 (12)	0.0235 (12)	0.0368 (14)	0.0002 (10)	−0.0031 (10)	0.0092 (10)
C6	0.0222 (12)	0.0245 (12)	0.0333 (13)	0.0051 (10)	0.0014 (10)	0.0116 (10)
C7	0.0213 (12)	0.0263 (13)	0.0388 (15)	0.0045 (10)	0.0009 (11)	0.0107 (11)
C8	0.0286 (14)	0.0294 (13)	0.0322 (14)	0.0021 (11)	−0.0030 (11)	0.0106 (11)
C9	0.0259 (13)	0.0275 (13)	0.0362 (14)	0.0068 (11)	0.0033 (11)	0.0124 (11)
C10	0.0466 (17)	0.0358 (16)	0.0341 (15)	0.0054 (13)	−0.0028 (12)	0.0083 (12)
C11	0.069 (2)	0.0491 (19)	0.0354 (16)	0.0176 (17)	−0.0004 (15)	0.0176 (14)
C12	0.076 (2)	0.0401 (17)	0.0501 (19)	0.0084 (17)	0.0044 (17)	0.0258 (15)
C13	0.0525 (18)	0.0347 (16)	0.0460 (18)	−0.0019 (14)	−0.0004 (14)	0.0158 (13)
C14	0.0352 (15)	0.0346 (15)	0.0347 (15)	0.0056 (12)	0.0037 (11)	0.0112 (12)
C15	0.0407 (16)	0.0376 (16)	0.0331 (15)	0.0033 (13)	−0.0006 (12)	0.0115 (12)
OW1	0.0400 (12)	0.0408 (11)	0.0403 (12)	0.0014 (9)	−0.0043 (9)	0.0192 (9)

### Geometric parameters (Å, º)

**Table:**

Cu1—O7^i^	1.9204 (18)	C2—C3	1.380 (3)
Cu1—O7	1.9204 (18)	C2—C7	1.495 (3)
Cu1—N1^i^	1.956 (2)	C3—C4	1.391 (3)
Cu1—N1	1.956 (2)	C3—H3	0.9300
Cu1—O3	2.837 (2)	C4—C5	1.388 (3)
O1—C7	1.203 (3)	C4—C8	1.479 (3)
O2—C7	1.313 (3)	C5—C6	1.385 (3)
O2—H2	0.8200	C5—H5	0.9300
O3—C8	1.246 (3)	C6—C9	1.497 (3)
O4—C8	1.281 (3)	C10—C11	1.379 (4)
O4—H4	0.8200	C10—H10	0.9300
O5—C9	1.210 (3)	C11—C12	1.373 (4)
O6—C9	1.303 (3)	C11—H11	0.9300
O6—H6	0.8200	C12—C13	1.369 (4)
O7—C15	1.278 (3)	C12—H12	0.9300
O8—C15	1.227 (3)	C13—C14	1.374 (4)
N1—C10	1.335 (3)	C13—H13	0.9300
N1—C14	1.347 (3)	C14—C15	1.505 (4)
C1—C6	1.385 (3)	OW1—HW1A	0.8499
C1—C2	1.390 (3)	OW1—HW1B	0.8499
C1—H1	0.9300		
			
O7^i^—Cu1—O7	180.0 (2)	C4—C5—H5	120.0
O7^i^—Cu1—N1^i^	84.32 (8)	C5—C6—C1	119.8 (2)
O7—Cu1—N1^i^	95.68 (8)	C5—C6—C9	118.4 (2)
O7^i^—Cu1—N1	95.68 (8)	C1—C6—C9	121.7 (2)
O7—Cu1—N1	84.32 (8)	O1—C7—O2	124.7 (2)
N1^i^—Cu1—N1	180.0	O1—C7—C2	123.2 (2)
O7^i^—Cu1—O3	99.58 (8)	O2—C7—C2	112.1 (2)
O7—Cu1—O3	80.42 (8)	O3—C8—O4	123.3 (2)
N1^i^—Cu1—O3	85.19 (8)	O3—C8—C4	120.2 (2)
N1—Cu1—O3	94.81 (8)	O4—C8—C4	116.4 (2)
C7—O2—H2	109.5	O5—C9—O6	124.5 (2)
C8—O3—Cu1	113.90 (18)	O5—C9—C6	120.8 (2)
C8—O4—H4	109.5	O6—C9—C6	114.7 (2)
C9—O6—H6	109.5	N1—C10—C11	121.5 (3)
C15—O7—Cu1	114.81 (17)	N1—C10—H10	119.2
C10—N1—C14	119.2 (2)	C11—C10—H10	119.2
C10—N1—Cu1	129.42 (19)	C12—C11—C10	119.1 (3)
C14—N1—Cu1	111.42 (18)	C12—C11—H11	120.5
C6—C1—C2	120.5 (2)	C10—C11—H11	120.5
C6—C1—H1	119.8	C13—C12—C11	119.6 (3)
C2—C1—H1	119.8	C13—C12—H12	120.2
C3—C2—C1	119.5 (2)	C11—C12—H12	120.2
C3—C2—C7	120.5 (2)	C12—C13—C14	119.0 (3)
C1—C2—C7	120.0 (2)	C12—C13—H13	120.5
C2—C3—C4	120.4 (2)	C14—C13—H13	120.5
C2—C3—H3	119.8	N1—C14—C13	121.6 (3)
C4—C3—H3	119.8	N1—C14—C15	114.3 (2)
C5—C4—C3	119.9 (2)	C13—C14—C15	124.1 (3)
C5—C4—C8	121.4 (2)	O8—C15—O7	125.4 (3)
C3—C4—C8	118.6 (2)	O8—C15—C14	119.5 (3)
C6—C5—C4	120.0 (2)	O7—C15—C14	115.1 (2)
C6—C5—H5	120.0	HW1A—OW1—HW1B	109.5
			
C6—C1—C2—C3	−0.7 (4)	C5—C6—C9—O5	−2.9 (4)
C6—C1—C2—C7	−178.8 (2)	C1—C6—C9—O5	−179.6 (2)
C1—C2—C3—C4	−0.8 (4)	C5—C6—C9—O6	175.6 (2)
C7—C2—C3—C4	177.3 (2)	C1—C6—C9—O6	−1.1 (4)
C2—C3—C4—C5	1.7 (4)	C14—N1—C10—C11	0.6 (4)
C2—C3—C4—C8	−174.1 (2)	Cu1—N1—C10—C11	179.5 (2)
C3—C4—C5—C6	−1.0 (4)	N1—C10—C11—C12	1.7 (5)
C8—C4—C5—C6	174.6 (2)	C10—C11—C12—C13	−2.1 (5)
C4—C5—C6—C1	−0.5 (4)	C11—C12—C13—C14	0.3 (5)
C4—C5—C6—C9	−177.2 (2)	C10—N1—C14—C13	−2.4 (4)
C2—C1—C6—C5	1.3 (4)	Cu1—N1—C14—C13	178.4 (2)
C2—C1—C6—C9	178.0 (2)	C10—N1—C14—C15	178.5 (2)
C3—C2—C7—O1	−177.5 (3)	Cu1—N1—C14—C15	−0.6 (3)
C1—C2—C7—O1	0.6 (4)	C12—C13—C14—N1	2.0 (5)
C3—C2—C7—O2	2.0 (3)	C12—C13—C14—C15	−179.1 (3)
C1—C2—C7—O2	−179.9 (2)	Cu1—O7—C15—O8	−177.8 (3)
Cu1—O3—C8—O4	113.7 (3)	Cu1—O7—C15—C14	2.5 (3)
Cu1—O3—C8—C4	−67.3 (3)	N1—C14—C15—O8	179.1 (3)
C5—C4—C8—O3	171.8 (3)	C13—C14—C15—O8	0.1 (5)
C3—C4—C8—O3	−12.5 (4)	N1—C14—C15—O7	−1.2 (4)
C5—C4—C8—O4	−9.2 (4)	C13—C14—C15—O7	179.7 (3)
C3—C4—C8—O4	166.5 (2)		

Symmetry code: (i) −*x*, −*y*, −*z*.

### Hydrogen-bond geometry (Å, º)

**Table:**

*D*—H···*A*	*D*—H	H···*A*	*D*···*A*	*D*—H···*A*
O2—H2···O5^ii^	0.82	1.84	2.621 (2)	158
O4—H4···O3^iii^	0.82	1.85	2.659 (3)	167
O6—H6···O*W*1^iv^	0.82	1.73	2.553 (3)	176
C10—H10···O7^i^	0.93	2.66	3.121 (3)	112
C13—H13···O4^v^	0.93	2.61	3.454 (4)	152
O*W*1—H*W*1*A*···O8^vi^	0.85	1.88	2.729 (3)	174
O*W*1—H*W*1*A*···O7^vi^	0.85	2.66	3.259 (3)	129
O*W*1—H*W*1*B*···O1	0.85	2.02	2.872 (3)	175

Symmetry codes: (i) −*x*, −*y*, −*z*; (ii) *x*−1, *y*−1, *z*; (iii) −*x*+1, −*y*, −*z*; (iv) *x*+1, *y*+1, *z*; (v) −*x*+1, −*y*+1, −*z*; (vi) *x*−1, *y*−1, *z*−1.
